# Adipositas and metabolic bone disorder in a 16th century Upper Austrian infant crypt mummy—An interdisciplinary palaeopathological insight into historical aristocratic life

**DOI:** 10.3389/fmed.2022.979670

**Published:** 2022-10-26

**Authors:** Andreas G. Nerlich, Stephanie Panzer, Judith Wimmer, Christian Hamann, Oliver K. Peschel

**Affiliations:** ^1^Institute of Pathology, Academic Clinic Munich-Bogenhausen, Munich, Germany; ^2^Department of Radiology, Trauma Centre Murnau, Germany and Institute of Biomechanics, Paracelsus Medical University, Salzburg, Austria; ^3^Department of Art and Heritage Conservation, Diocese of Linz, Linz, Austria; ^4^Leibniz-Laboratory for Age Estimation, Christian-Albrecht-University, Kiel, Germany; ^5^Institute of Legal Medicine, Ludwig-Maximilians University, Munich, Germany

**Keywords:** palaeopathology, vitamin D deficiency, rickets, adipositas, pneumonia, pseudopathology

## Abstract

We describe here the results of a multidisciplinary study on an infant mummy from 16th century Upper Austria buried in the crypt of the family of the Counts of Starhemberg. The macroscopic-anthropological, radiological (whole-body CT scan), histological (skin tissue), and radiocarbon isotope investigations suggested a male infant of 10–18 months' age, most likely dying between 1550 and 1635 CE (probably Reichard Wilhelm, 1625–1626 CE), that presented with evidence of metabolic bone disease with significant bilateral flaring of costochondral joints resembling “rachitic rosary” of the ribs, along with straight long bones and lack of fractures or subperiosteal bleeding residues. Although incompletely developed, the osteopathology points toward rickets, without upper or lower extremities long bone deformation. The differential diagnosis is vitamin C deficiency (scurvy) (also with an incomplete presentation, although overlap between both disorders may be present). As additional pathology, there was significantly enlarged subcutaneous fat tissue (thickness more than 1 cm at the navel and thighs and longitudinal creases of the skin) along with a histologically enlarged subcutaneous fat layer consistent with infantile adipositas as a coincident disorder. Finally, remnants of lung tissue with pleural adhesion of the right lung indicate possibly lethal pneumonia, a disease with an increased prevalence in vitamin D deficient infants. Ultimately, the skull presented with extensive destruction of the bones of the base and dislocation of the bones of the skull squama. These changes, however, are most likely post-mortal pseudopathology, the result of a burial in a flat, narrow coffin because there were no bone fractures or residues of bleeding/tissue reaction that would have occurred whilst the patient was alive.

## Introduction

Whilst recent anthropological and palaeopathological examination of human remains of past populations provides more and more insight into living conditions, disease, and possibly the cause of death in historic populations, the information on infants and their fate is comparably sparse. This is mostly due to the fact that the preservation of infantile human remains is frequently limited due to their smaller size, the significantly higher fragility of the biomaterial, and less care during material recovery (1, 2). Accordingly, standard physical anthropological textbooks have coined the term “infant deficiency” in most historical settings (3, 4), indicating a “loss” of material and the resulting data. Whilst soft tissue and bone of infants may perish very rapidly and completely in human soil burials, bodies with artificial or spontaneous mummification and protected storage conditions, such as in crypt burials, may result in preservation. These rare findings provide unique insight into the infant's life (5, 6).

Until now, there exist only isolated case reports or small series on infant mummies from areas with a cultural history of embalming or non-intentional mummification, such as in ancient Egypt and South America; only rare cases of mummified infants have been described from European locations. Our interdisciplinary study reports another case of a well-preserved aristocratic infant mummy from the 1600s CE. The little body survived into present times due to the fact that it was a member of a high aristocratic family with a burial in a protective crypt setting. This study provides clear evidence for infantile palaeopathology which may have partly escaped from detection in cases with only preserved skeletons.

The main aim of the study was to obtain relevant information about the potential identification of the infant. The secondary aim was to identify the status and nature of its tissue preservation and any measures necessary for the maintenance of the corpse.

## Materials and methods

### The infant

The naturally mummified body of the infant comes from the family crypt of the Counts of Starhemberg, one of the oldest aristocratic families in Austria. It is located close to the family residence at Wildberg castle, in the small village of Hellmonsödt, Upper Austria. This is some 15 km north of the Upper Austrian capital Linz in the mountain region of the “Mühlviertel” (7, 8). The church, built in 1441 CE, was extended in 1499 CE by adding a memorial chapel with a crypt underneath, accommodating the burials of numerous members of the Starhemberg family in beautifully decorated metal coffins with inscriptions, and one small wooden coffin without an indication of its content or origin (8, 9). Accordingly, there exists no direct evidence of the infant's name or other information. It is the only infant mummy in the crypt.

### Ethical and juridical permissions

The study was approved by the Diocese of Linz, Upper Austria. Additionally, oral consent was obtained from the local church authorities and the head of the still-existing family branch.

### Macroscopic and radiological examination

During some restoration work on the crypt, the opportunity was taken to open the infant's coffin and a macroscopic investigation was undertaken. The body underwent anthropological measurements as far as possible and, subsequently, submitted to a whole-body CT scan. The scan (64-row detector, Siemens Somatom Scope, Siemens Healthcare, Erlangen, Germany) was performed in the supine position with a slice thickness of 0.625 mm, an interval of 0.625 mm, 120 kV, and 200 mA in the standard algorithm as previously described (10, 11). Additional three-dimensional and multi-planar reconstructions were prepared on a workstation (Siemens Healthcare, Erlangen, Germany). This was particularly applied to the reconstruction of the skull. Certain reconstructed skeletal elements (femur, tibia, and humerus) were additionally used to calculate bone length as part of the anthropological evaluation.

### Radiocarbon analysis

Further relevant data were obtained from a soft tissue biopsy for radiocarbon dating and histological examination. A small piece (c. 6 x 2 mm) of skin/subcutaneous soft tissue was removed from the lower lumbar region of the mummy with a scalpel. The material was divided into two; a larger piece for radiocarbon dating and a smaller one for histology.

The radiocarbon dating was performed after the extraction of skin protein (mainly collagen) according to routine protocols (12). Thereby, 3.2 mg material could be analyzed by accelerator mass spectrometry (AMS) at the Leibniz-Laboratory for Radiometric Dating and Isotope Research, Kiel University (Germany) (AMS Type HVE 3MV Tandetron 4130) using the calibration data by Reimers et al. (13).

### Histological examination

The histological analysis was prepared with a rehydration procedure, followed by the embedding and cutting as previously described in detail (14). The prepared sample was serially sectioned (2–3 μm thickness) and then stained with H&E, connective tissue stains, PAS, Grockotts' silver stain, and Prussian blue (14).

## Results

### Macroscopic and anthropological examination

**Figure 1:**
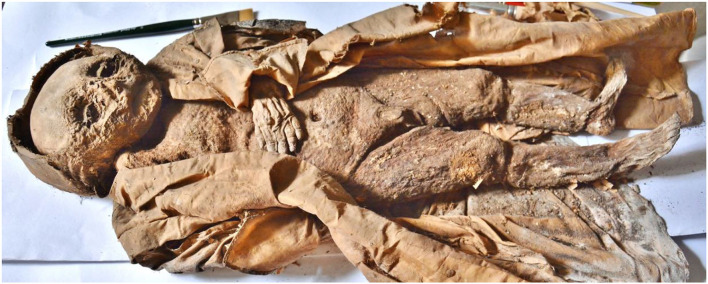
The infant mummy of the Hellmonsödt crypt. Overview of the complete body with the silk coat.

The male infant was found enveloped in a very elaborate long silk coat which included a hood covering the skull. In parallel with this anthropological–paleopathological investigation, the coffin and coat of the infant were investigated and restored by the Department of Art and Heritage Conservation, Diocese of Linz, Austria (E. Biegler-Machow and JW). Whilst the coffin did not provide further useful information, the coat analysis showed a socially high-status fabric made of perfectly woven silk which was excellently preserved. The body was lying in the supine position, the right arm along the right side of the body, the left arm angled in the elbow joint with the left hand resting on the upper abdomen (Figure 1).

**Figure 2:**
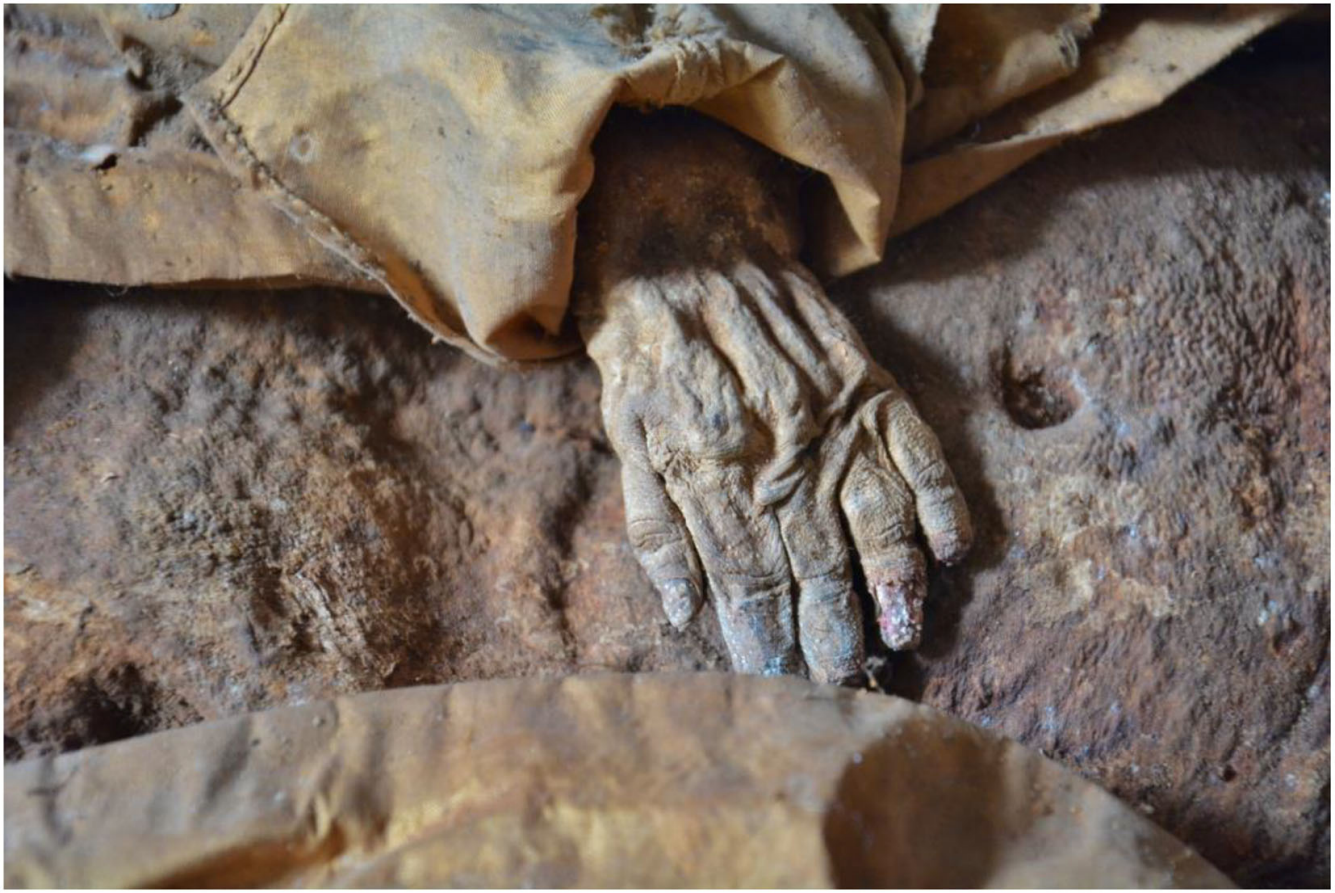
Detail of the mummy: left hand on the abdomen. Note also the retracted navel.

The skin of the ventral, dorso-thoracic, and abdominal walls was completely intact without any evidence of incisions or other manipulations. It was considerably darkened, but otherwise very well preserved with, for instance, the finger and toe nails were intact. The umbilicus was significantly retracted (Figure 2) where the thickness of the abdominal wall was measured as approximately 1 cm. The soft tissues of the abdomen and both thighs contained longitudinal creases suggesting a much more voluminous thickness of the soft tissues prior to death. There were no obvious malformations of the post-cranium. The external genitalia were clearly male. The urethral and anal orifices were unremarkable.

**Figure 3:**
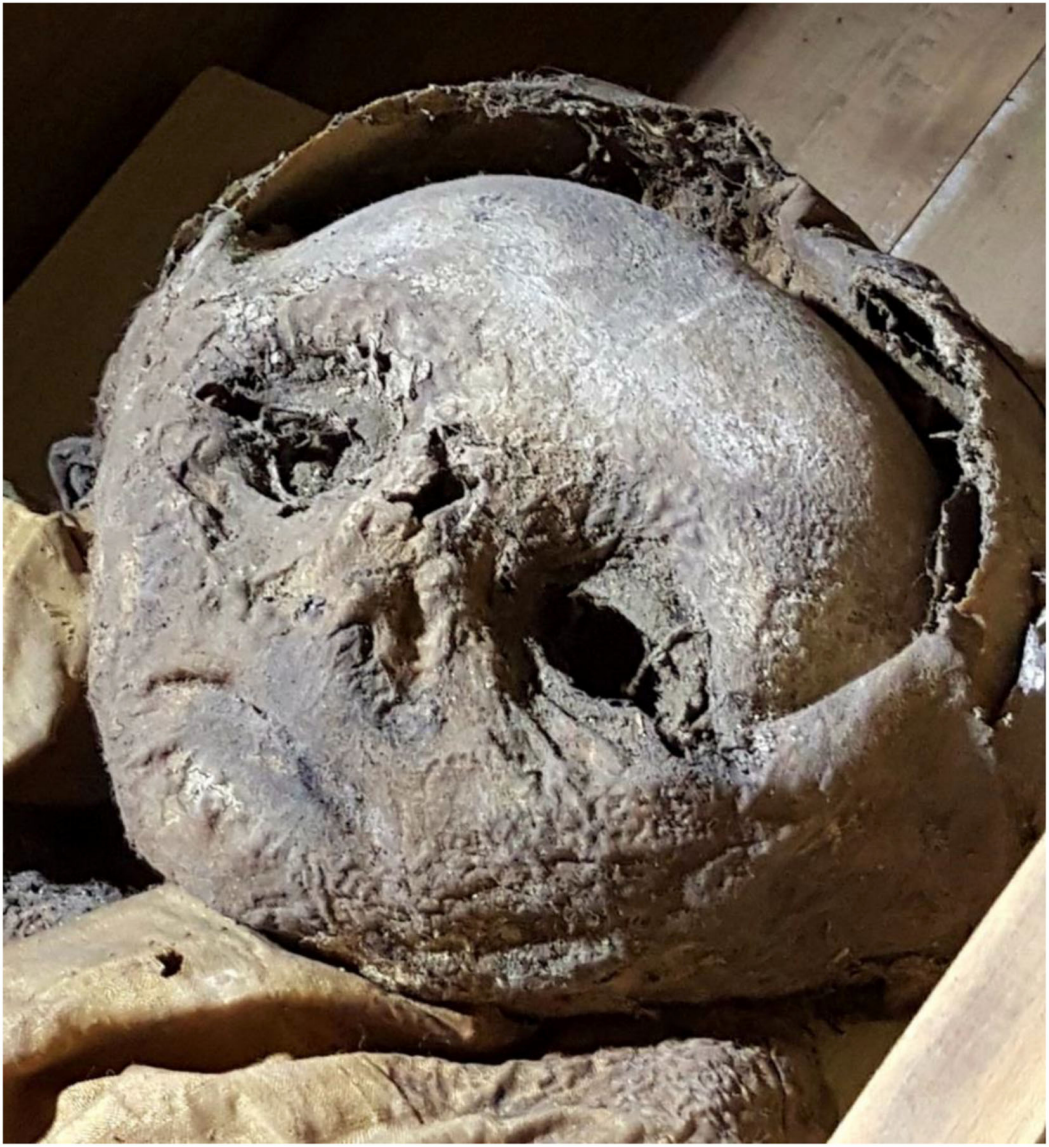
Detail of the mummy's face. Note here the defects of the skin at the chin and nose and the gap between the frontal bone and the silk hood.

In contrast to the post-cranium, the face and skull appeared abnormal in that the face seemed to be flattened, with several small skin defects at the chin and nose (Figure 3). Furthermore, there was a gap between the calvarium and the hood of the silk cape. The eyelids were closed and slightly retracted and the eyes seemed to be significantly shrunken. The nasal orifices were regularly formed. The ears were hidden by the silk hood.

The body had a crown-to-heel length of 53 cm. Due to the facial deformity, no further measurements were taken from the skull.

### CT scan

**Figure 4:**
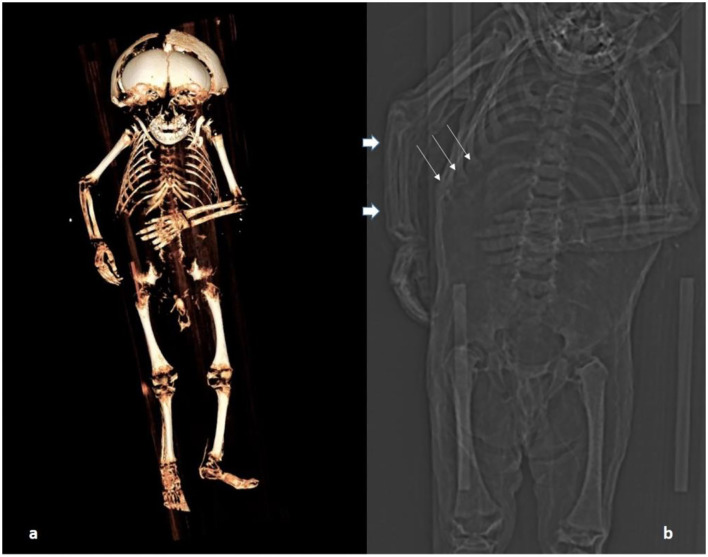
CT scan of the body: **(a)** Three-dimensional reconstruction of the skeleton. **(b)** A section of the topogram showing particularly the rosary of the costochondral junction (thin arrows); long bones are straight (possibly the right ulna is very slightly bent; thick arrows) and the metaphyses are minimally enlarged at upper and lower long bones.

The whole-body scan produced 613 axial slices which were also used for subsequent three-dimensional reconstructions (Figure 4). The images confirmed the excellent state of preservation of the mummy including bone and soft tissue structures.

### Evaluation of bone measurements

The various skeletal elements of the post-cranium were anatomically aligned. Typical ossification centers were seen at the epiphyses of the long bones, but were absent from small bones of the hand, wrist, and feet, indicating an individual aged between 12 and 18 months old (2, 15, 16). Individual long bones measured: right femur 113 mm, left femur 115 mm, right tibia 95 mm, left tibia 96 mm, right humerus 92 mm, and left humerus 96 mm. These measurements indicate an individual aged between 10 and 14 months old [regression calculation according to Cardoso et al. (17)]. Finally, since the mandible and maxilla were intact, the position of the teeth with the eruption of the incisors also indicated an individual aged between 1 year and 1.5 years old (18). These age estimations were used for a consensus age evaluation of the individual ranging between 10 and 18 months.

### Palaeopathology of the post-cranial skeleton

**Figure 5:**
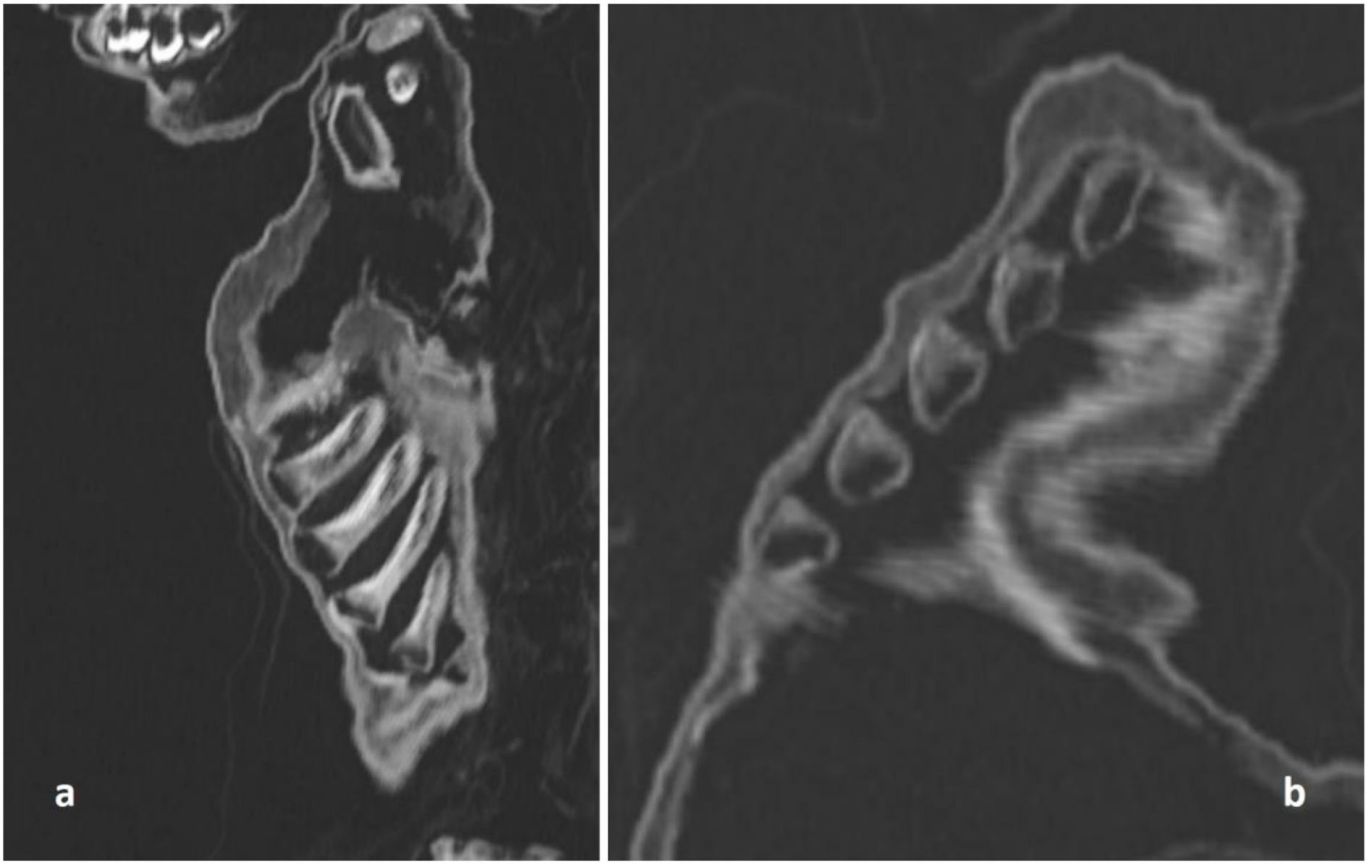
CT scan of the costochondral junction with an enlargement such as seen in “rickety rosary” but also in “scurvy rosary.” **(a)** Sagittal section; **(b)** oblique section.

The post-cranial skeleton showed distinct pathology. The costochondral joints of the rib cage had bilateral knob-like expansion (Figure 5a). These changes affected all the costochondral joints and were even more pronounced in oblique CT sections (Figure 5b). The epi-metaphyses of long bones appeared only slightly enlarged. However, other typical features of rickets were absent. The long bones, both of upper and lower extremities, were neither significantly bowed nor otherwise malformed. Only the right ulna might have been slightly bent (Figure 4b). There were no fractures. The radiodensity of the bones did not appear abnormal. The joints and flat bones were unremarkable apart from the epi-metaphyseal growth plates which were slightly enlarged. Finally, there was no evidence for periosteal bone apposition nor are any signs of destruction at the growth plate, e.g., Looser or “Trümmerzone” changes (19).

### Evaluation of the skull bones

**Figure 6:**
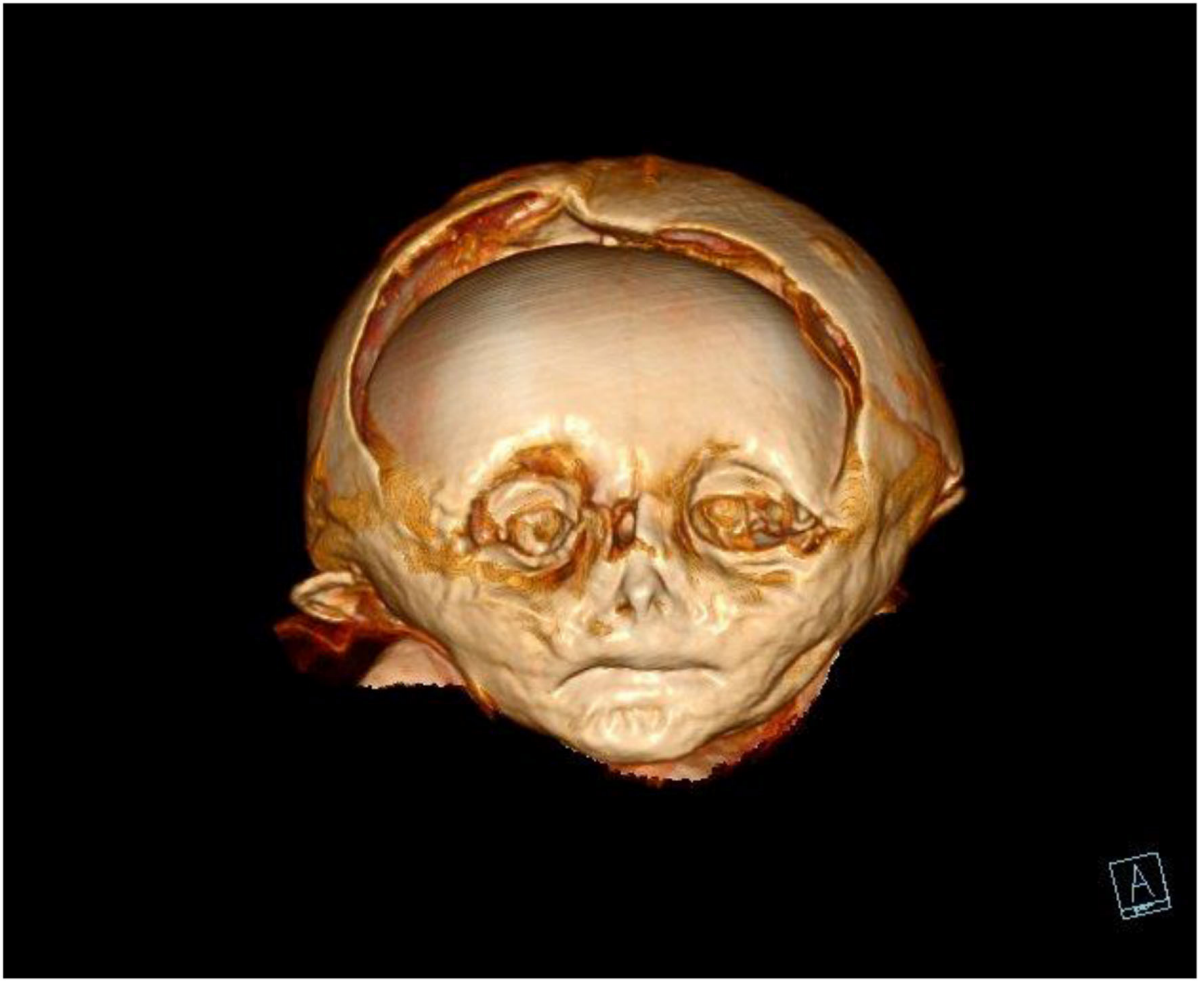
CT scan of the mummy's head. On three-dimensional reconstruction, the deformation of the skull bones, especially of the frontal and the parietal squamae are seen.

**Figure 7:**
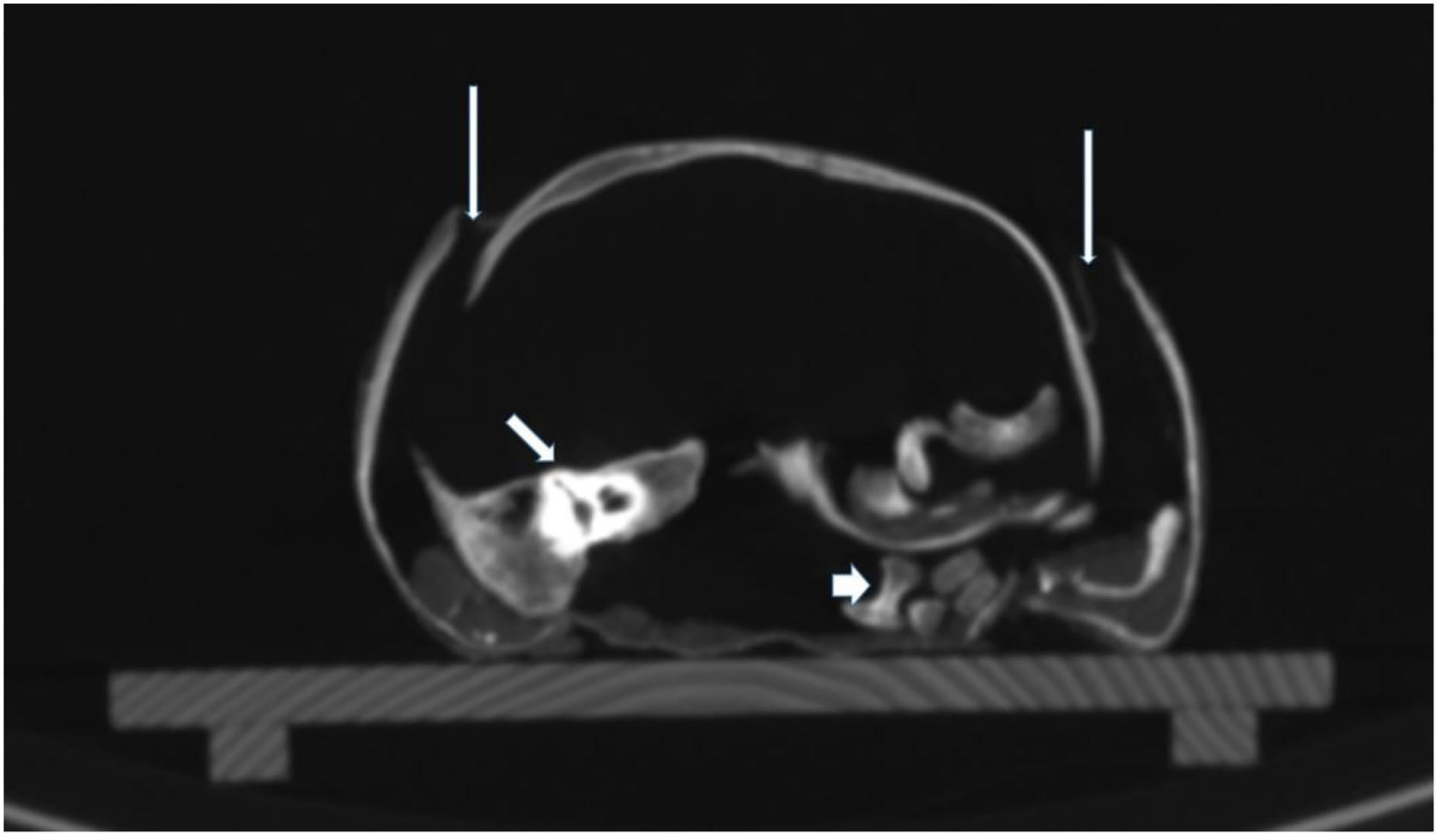
CT scan of the skull base. On cross-sections, the extensive dislocation of the bones of the skull base is visible. Thin arrows: disruption of sutures; middle arrow: dislocated right petrous bone; thick arrow: dislocated parts of vertebral bodies.

The skull was morphologically remarkable with an unusually flat face and skin defects (Figure 3). On 3-D reconstruction, a dislocation of most bones was seen (Figure 6). The skull base was similarly affected including the upper three cervical vertebral bodies. These were disrupted and irregularly arranged (Figure 7), although most of the dislocation in its present state may have come from a collapse of the skull bone integrity. In contrast, the mid-face, including the maxilla, the mandible, and the lower cervical spine, appeared normal and the joints were not dislocated. The coronal suture was disrupted and the frontal squama was shifted below the ventral parts of the parietal squamae which seemed closely attached to the covering silk hood (Figure 6). Taken together, the bone dislocations showed major abnormalities which might have been the result of severe intra-vital trauma or an extensive post-mortem dislocation. There were no signs of bone fractures, bone remodeling, bleeding residues, or any signs of healing.

### Anatomical and palaeopathological findings of the soft tissues and inner organs

**Figure 8:**
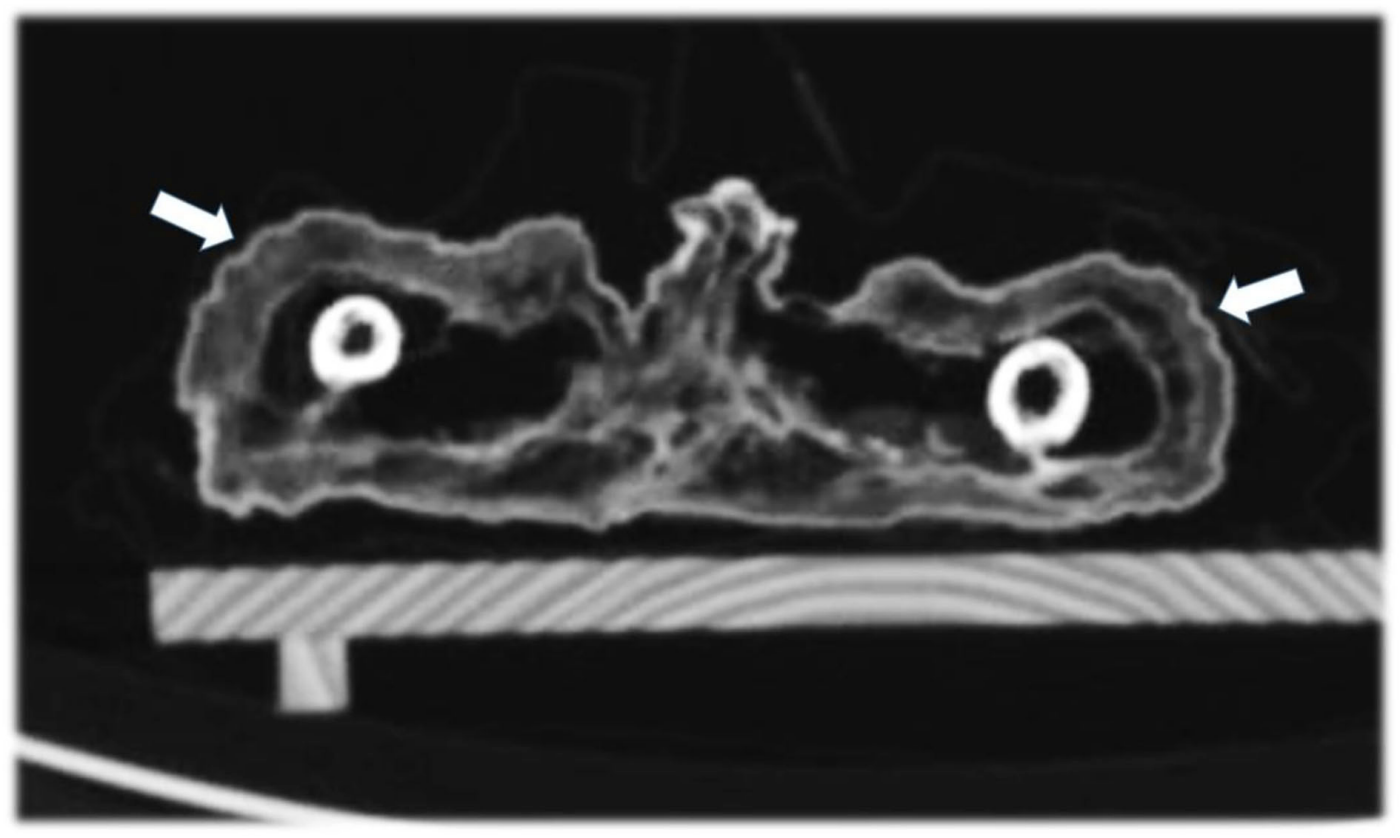
Axial CT sections through the thighs showing the broad subcutaneous fat tissue envelope of the mummy (arrows).

**Figure 9:**
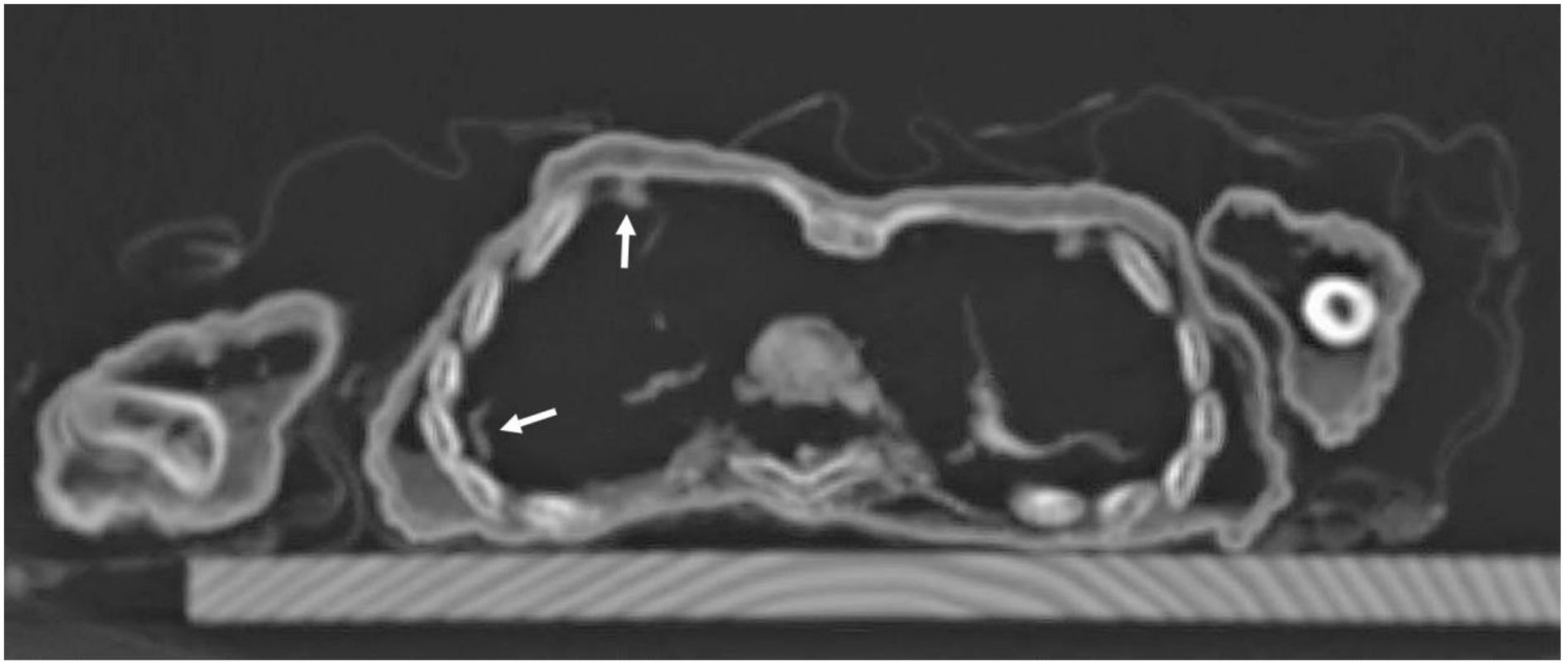
Axial CT sections through the chest: besides the regular skeletal elements, there are remnants of lung tissue in the right chest cavity with some pleural adhesions (arrows).

The soft tissue of the subcutis was remarkable in that it seems considerably thickened. This was most evident at the umbilicus which was retracted by approximately 1 cm. This strongly suggested significant fat tissue accumulation (Figure 8). A further evaluation of the internal organs showed remains of the lungs which spanned the right chest cavity (Figure 9). Additionally, the pericardium was seen *in situ*, but no myocardial remains could be identified. In the upper abdomen, the remains of the liver and, remarkably, the intestinal loops that appeared expanded and fixed in a net-like arrangement, were present. These organ remnants were anatomically located and without obvious pathological findings.

### Radiocarbon dating

**Figure 10:**
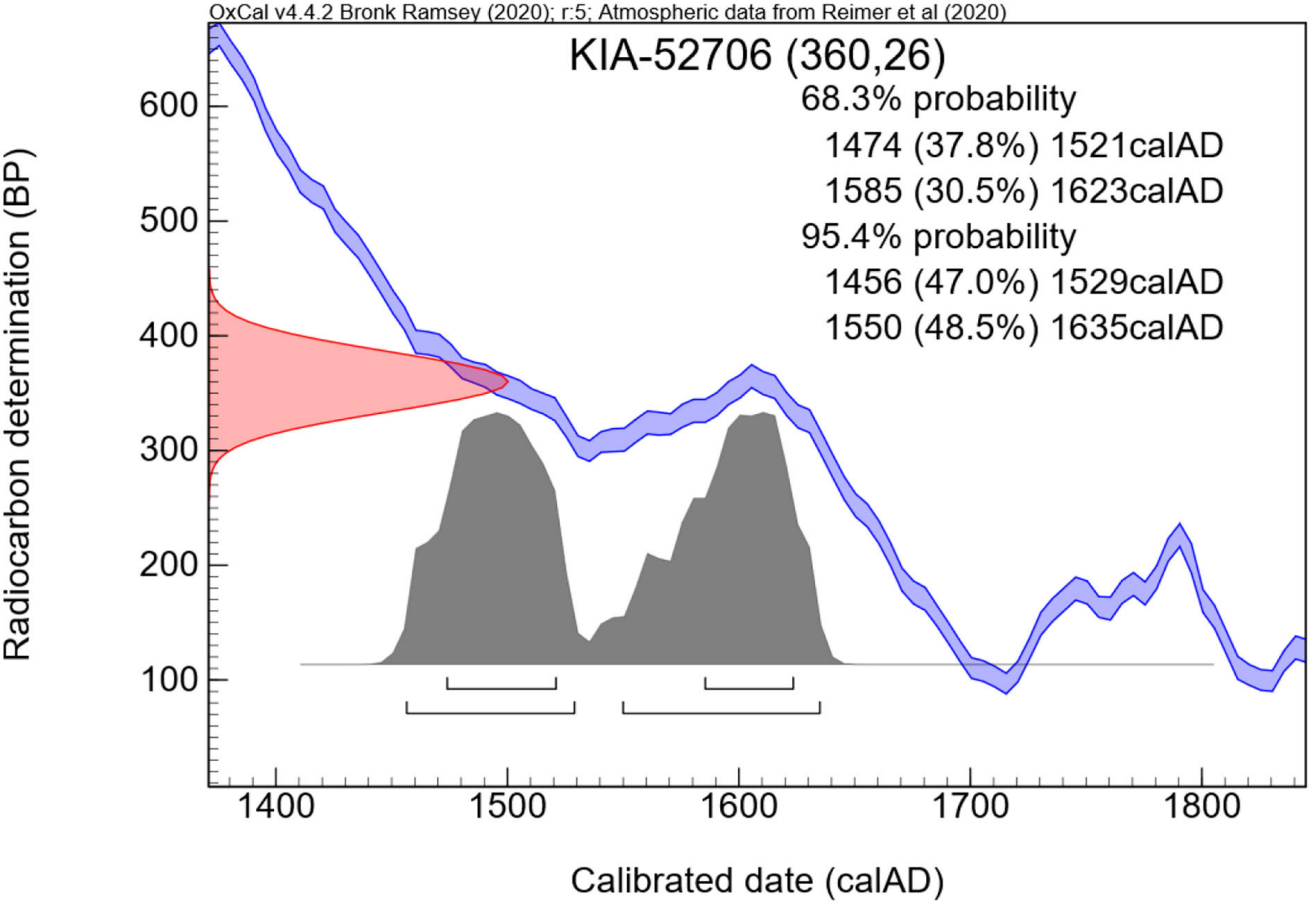
Result of the radiocarbon dating. Note the calibration zone that determines the calculated time of individual death.

Radiocarbon dating showed two possible time segments of the calibration curve within the 95.4% probability range. The measurements gave 360 +/— 26 years before present which means a calibrated death time period either between 1456–1529 CE or 1550–1635 CE (Figure 10).

### Histological skin soft tissue examination

**Figure 11:**
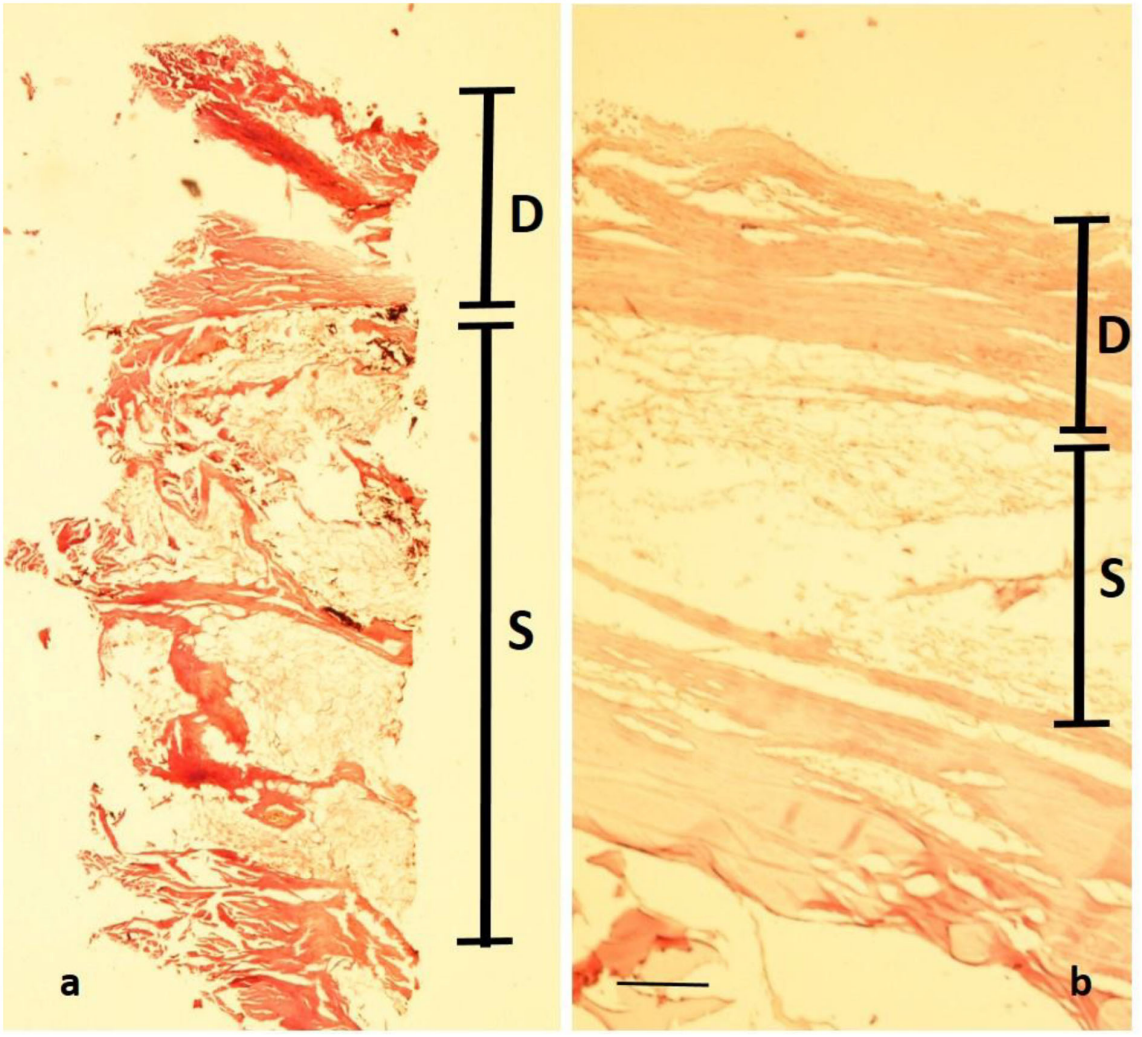
Histopathology of the mummy's skin following rehydration and embedding: although the epidermis has been lost, a well-preserved dermal collagenous connective tissue is seen (“D”) which is followed by subcutaneous fat tissue (“S”). **(a)** Cross-section of the mummy sample; **(b)** similar samples (age- and site-matched) from an aristocratic infant mummy from South Germany (Staining: H&E; bar: 500 μm).

**Figure 12:**
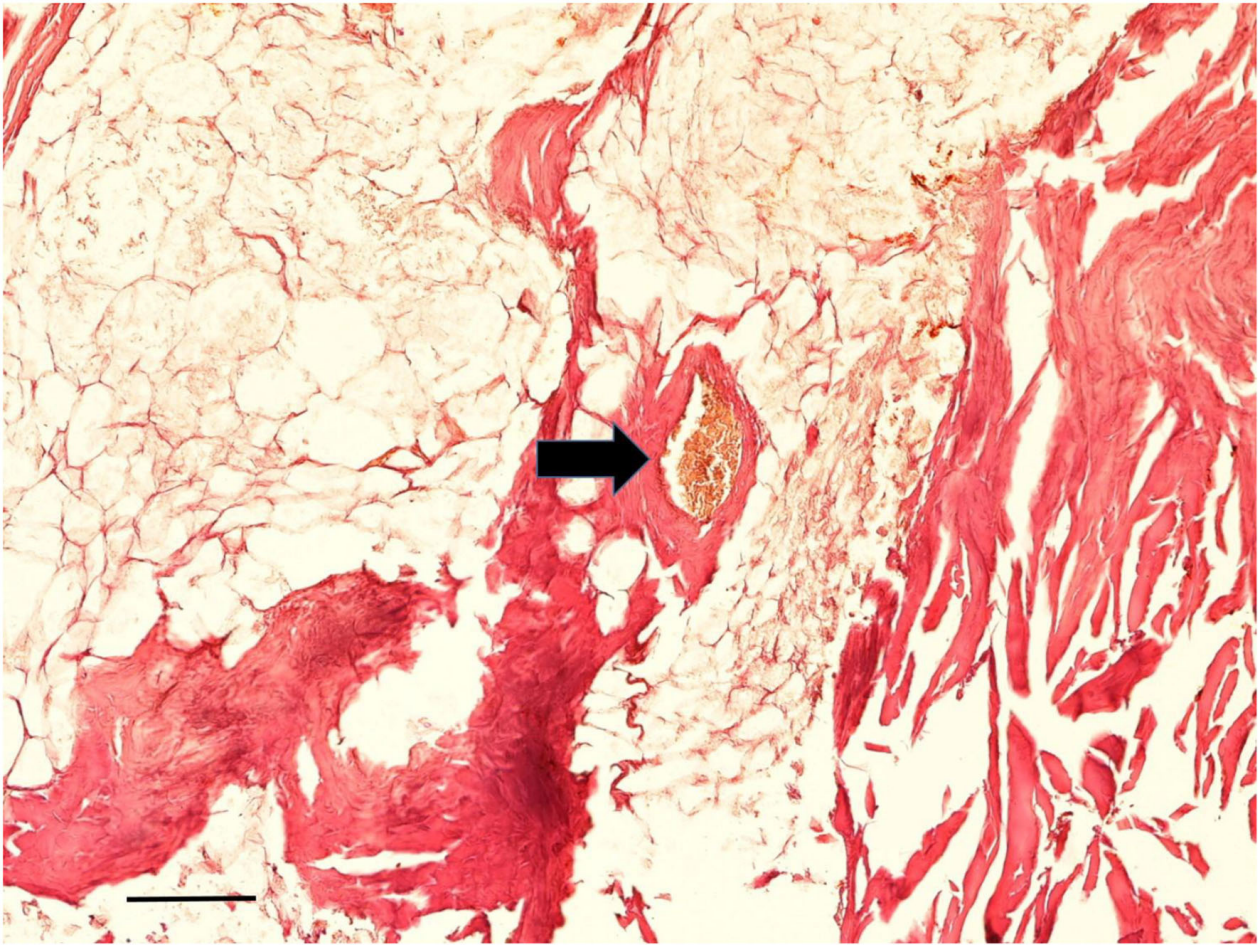
Histological examination of subcutaneous tissue. Subcutaneous fat tissue with little effects of adipocere transformation is seen together with typical small blood vessels (arrow) (Staining: H&E; bar: 100 μm).

**Figure 13:**
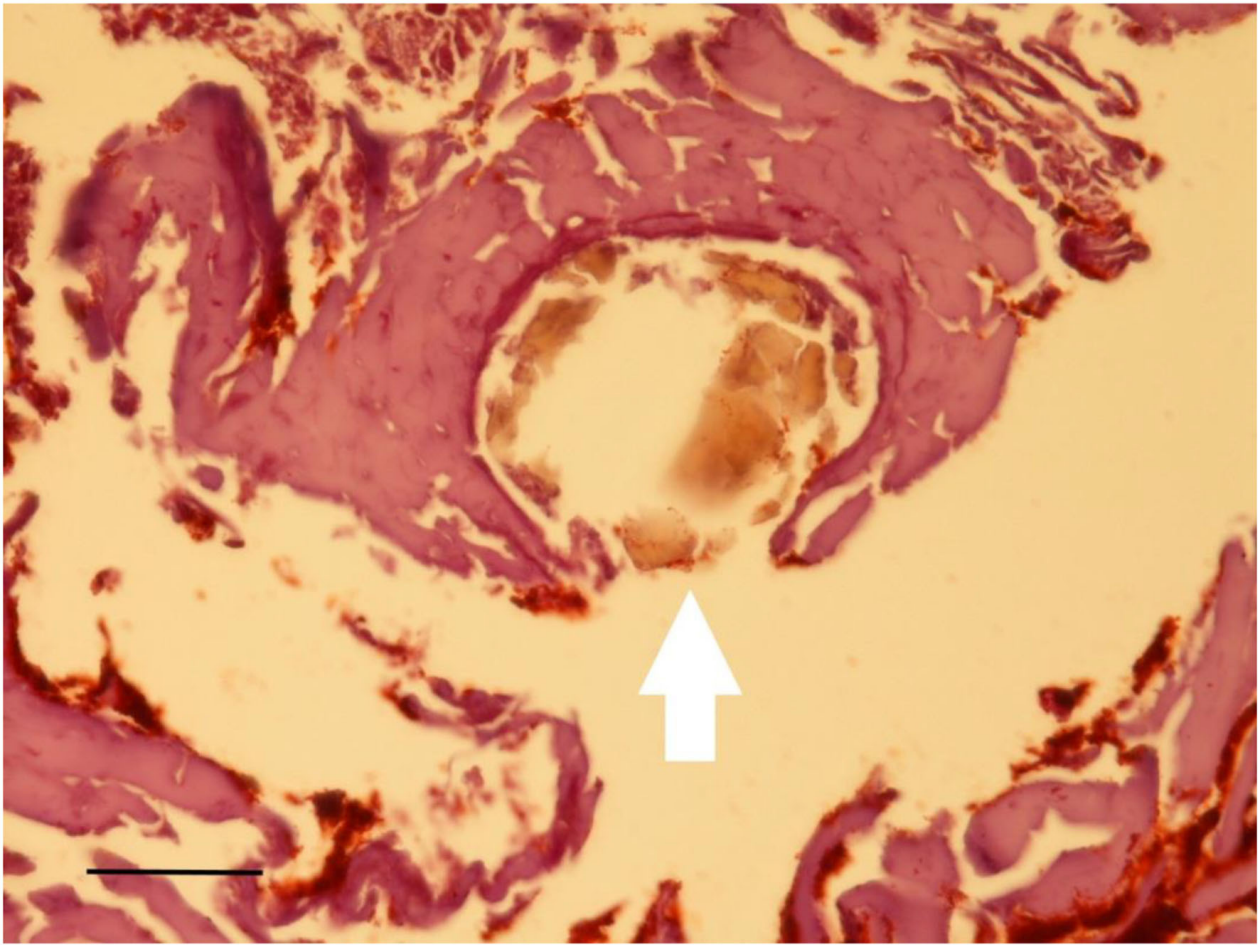
Histological examination of the subcutaneous skin sample. Occasional inclusion of a hair shaft with dark pigmentation (arrow) (Staining: H&E; bar: 25 μm).

A very small full-thickness sample of the skin biopsy was used for histopathological evaluation. The superficial epidermis was absent, as expected. However, the subcutaneous collagenous soft tissue was excellently preserved showing the very typical woven bundles. Seen interspersed were islands of monovacuolar fat cells which were significantly enlarged, particularly when compared to an age- and site-matched control case (Figure 11). Accordingly, the subcutaneous fat pad was nearly double in size of the control. Focally, slight effects of adipocere transformation were seen (Figure 12). In summary, the extent of the fat tissue significantly exceeded that of the normal level. Further histoanatomical structures, such as small subcutaneous blood vessels and occasional skin appendages, and hair shafts, were retained in excellent condition. All structures were non-pathological. One of the hair follicles was dark colored, suggesting dark colouration of the infant's hair (Figure 13).

## Discussion

The life and living conditions of infants in historical populations have only been investigated to a limited extent. The main reason is the lack of study material mainly due to the rapid degradation of infantile human remains following burials and the extreme rarity of the practice of embalming infants and preservation of the mummies (3, 4). Despite the known high infant death rates, the information on those infants' lives frequently comes from literary sources with all the problems of the literary tradition for factual information (20). Therefore, the meticulous investigation of infant mummies offers an additional source of data.

In populations that practiced artificial embalming, more cases, or even small case series, of infant mummy analyses are available, such as in Egypt (21–26) and South America (27, 28). Infant mummies from Europe have been available much more rarely (29–36). This seems to be due to the scarce practice of artificial embalming in Europe. In most instances, infant mummies that survived from Central Europe until today were spontaneously mummified. Accordingly, those burials in crypts are more relevant than the usual soil burials. As a consequence, aristocratic infant burials are more likely to have suitable infant remains than those of the lower social classes.

In this report, we describe one of those rare cases where spontaneous mummification of an infant has occurred found in an aristocratic family crypt. This was in a small town in Upper Austria belonging to the Starhemberg family, who lived close by. They are one of the oldest and most renowned aristocratic families in Austria tracing back to the 11th century (7). Wildberg castle was the family's home since at least the year 1212 CE. The nearby parish of Hellmonsödt was consequently used for burials by the family; initially as typical soil burials close to the church wall (8). Around the year 1500 CE, a crypt was built to house them. Since then, selected family members were entombed in this crypt. It underwent renovation around 1600 CE. Most of the cadavers were placed in beautifully decorated coffins that also recorded the names of the deceased. Accordingly, it is clear that the crypt mainly housed the burials of the title-holding (mostly first-born) males, and occasionally their wives (9). Unfortunately, with respect to the isolated infant burial, no inscription was placed on the wooden coffin and the plain unadorned coffin did not reveal the identity of the individual. Despite this, the infant's very expensive silk funerary coat is a clear indication that the body belonged to a family member of this aristocratic family.

### Information about the child's identity

As a first aim, this study was established to hopefully identify the individual. Besides the lack of direct written evidence from the coffin, the parish records from Hellmonsödt do not start before 1659 CE (at least, no older records have survived until today); therefore, contemporaneous documentation of the identity was not possible.

We, therefore, performed radiocarbon dating of a small subcutaneous sample which indicated the periods of death between 1456 and 1529 CE or 1550 and 1635 CE. This relatively large span of dates comes from the flat curve of the calibration dataset that is established to convert the non-calibrated measurements (here 360 +/– 26 years before present, i.e., before 1950 CE) into the expected span of death period. Since, however, the building of the crypt from the year 1499 CE was modified in the late 16th or beginning of the 17th century, the first range of data seems very unlikely since the cadaver would have to have been kept at another adequate burial place until the crypt was ready. There is no indication that the respective branch of the Starhemberg family had another family crypt at (or before) that time period. Accordingly, the infant most probably died between 1550 and 1635 CE. In addition, the first burial of an adult, Reichard von Starhemberg, in the reconstructed crypt took place in 1613 CE (9).

Furthermore, the body is clearly a male with an age at death ranging between 10 and 18 months as determined by several parameters (15). It is associated with the Starhemberg family. The well-documented family tree reveals that the potential number of individuals is very few (7, 8). Given that the burial crypt was restricted to family members, the radiocarbon dating period and the estimated age at death of 10–18 months indicate that there are 20 infants of the Starhembergs' to consider. Presuming that only first-born male infants were buried in the crypt as applied to the other burials (9), only three individuals remain. First, Gundaker, who was born in 1589 CE and died in 1590 CE, was the first-born boy to the stirps of Georg Achatz von Starhemberg (1559–1597 CE) and his wife Elisabeth von Schärffenberg (died 1600 CE). Second, Gregor, born in 1566 CE and died in 1567 CE, son of Heinrich von Starhemberg (1540–1574 CE) and his wife Maria Magdalena von Lamberg (died 1581 CE), provided that he was, indeed, the first-born infant although this has recently been debated (37). Finally, Reichard Wilhelm (1625–1626 CE), the first son of Erasmus der Jüngere (1595–1664 CE) and grandson of Reichard, the first entombed adult 13 years before, can be considered. Given that the crypt was renovated around 1600 CE and was used from 1613 to 1825 CE, Reichard Wilhelm is the boy who best fits the evidence.

### Palaeopathology of the infant

The comprehensive analysis of the body provided not only potential biographical data but also showed pathological abnormalities. The first of these was the evidence of obesity in the infant as demonstrated by the thickness of the subcutaneous fat, best evidenced at the umbilicus, where the fat thickness reached almost 1 cm. Further evidence for enhanced body fat mass came from the sizeable creases of the skin at both thighs. Both macroscopic features were confirmed by the CT scans and the histological analysis of significantly enhanced fat tissue at the subcutaneous level. The latter is shown in Figure 10, especially when compared to an age- and site-matched control obtained from a South German 1 $\frac{1}{4}$-years-old mummy from an aristocratic Bavarian family (38). The direct comparison of these two comparative samples indicated an almost doubling of the amount of fat in the mummy's subcutis. Although the modern-day criteria of “adipositas” may not be fulfilled (the body weight cannot be evaluated in this palaeopathological context), we suggest that this child exceeded any normal level of fat tissue. Furthermore, the presence of an enhanced fat layer indicates hyper-caloric nourishment in line with a high-class aristocratic status. Deposition of fat in internal organs was not seen, which might have especially being detected affecting the liver, particularly in a case of a congenital metabolic disease of fat metabolism, such as lipodystrophies or lipidoses (39). The formation of an adipocere suggests rapid burial and immediate hermetic air sealing with distinct storage conditions. According to Aufderheide (5), the adipocere transformation represents a particular decomposition-induced lipid modification under wet and air-tight conditions (such as in coffins, underwater, or within, for example, a glacier). However, it is fair to assume that the enhanced adipose fat tissue prevented the body from rapid decay (along with the immediate air exclusion) thereby contributing to the excellent preservation of the mummy.

The second metabolic disorder is primarily suggested by the significant chondro-osseous changes in the ribs, the “rosary” of the complete rib cage. Here, the osteochondral transition zone showed significant beaker-like, clearly pathological, enlargement. Whilst rickets is the most likely cause of this change, other typical signs, such as the long bone bowing, particularly of the femora and tibiae, were not present. Additionally, the typical cranial changes in rickets could not be evaluated in this case since the skull bones were severely disrupted.

Whilst we can, therefore, clearly diagnose a metabolic disorder of bone, there remain uncertainties as to the specific cause. It is possible that there is an overlap of causes as has previously been described in rickets and scurvy (19). Accordingly, rickets may be taken into consideration with a typical “rickety rosary”, but with an absence of major metaphyseal widening and long bone bowing. Since the latter develops only under mechanical loading, both bowing of arm and/or leg bones may be absent when the infant neither walks nor crawls (19). Furthermore, long bone bowing may be absent in the mild or the hyperplastic forms of rickets (the latter representing a vitamin D deficiency, but not a calcium deficiency) (19, 40). Alternatively, in severe vitamin C deficiency (scurvy), a rosary may occur (19). However, in our infant, additional signs of scurvy, such as subperiosteal ossification, fractures, or “Tümmerzone” changes in the epiphyseal growth plate were lacking. Finally, the infant may have suffered from hypophosphataemia, a very rare X-linked congenital disease that is defined by mutations in the phosphate metabolism leading to progressive skeletal deformities resembling typical vitamin D-deficient rickets (41, 42). Interestingly, affected individuals have a high rate of muscular weakness, which affects not only walking and crawling, but also breathing, and there are reports of an association with increased obesity (41). However, this disorder is extremely rare and there is no evidence of a familial increase in genetic disorders of the Starhemberg family, as far as available records indicate, though the child could have had a spontaneous mutation. Additionally, we were not permitted to obtain larger tissue samples and therefore cannot confirm with genome sequencing potential genetic mutations that would confirm this diagnosis.

Further information, on a total of nine individuals of the high aristocratic family of the Medici, Florence, Italy, comes from a previous palaeopathological study by Giuffra et al. (43) who examined only skeletal material. Out of the nine cases, four were of a similar age range to our infant (6–24 months). In some cases, only part of the skeleton could be evaluated. Only one of the two cases presented with rib flaring, two of three with bowed leg bones, and three of four cases with bowed arm bones. This highly important study accordingly supports the view that not all cases show all the signs of rickets.

In summary, our primary diagnosis is rickets. Because of its rarity, hypophosphataemia is less likely. The secondary diagnosis is scurvy, whilst other potential generalized bone disorders, such as connatal syphilis, can be excluded. Furthermore, localized bone diseases, such as post-trauma, inflammation, tumor, or hamartoma, are excluded. Similarly, localized costochondritis, especially of infectious origin due to the described post-pneumonic pleurisy (see below) can be ruled out, since the costochondral junctions of both sides of the thorax were similarly affected. In accordance with the Istanbul terminological framework in palaeopathology (44) where this case can be considered consistent with rickets, we suggest that rickets is the diagnosis that best fits the pathology described.

Rickets is the consequence of a lack of vitamin D, a vitamin that is first intestinally processed from pro-precursors, then further modified in the skin under the non-enzymatic action of sunlight to precursors and then further modified in the kidney to the active vitamin. The presence of sufficient vitamin D is required to mineralise the non-mineralised osteoid bone matrix into typical bone. Therefore, any interference of this pathway may result in a lack of vitamin D, and result in rickets in the growing skeleton. It was described first by Daniel Whistler (1619–1684 CE) in 1645 CE, rapidly followed by the description by Francis Glisson (1597–1677 CE) in 1651 CE. The latter had already attributed the disease to nutritional causes (45). The typical features of the disease, however, had already been presented pictorially in earlier paintings; notably by the German painter Hans Burgkmair the Elder (1473–1531 CE) in his canvas of “Virgin and Child” from 1509 CE where a rachitic rib rosary and bowing of the long bones are shown in the Child (46).

Vitamin C deficiency (scurvy) was first described clinically by Möller in 1859 CE and Barlow in 1883 CE (47, 48). However, the disease had long been identified as a distinct disease entity, for instance by Lind in 1753 CE (49). The main symptom is non-skeletal; bleeding of mucous membranes and the skin. Bleeding into the subperiosteal space is frequently seen which may result in the typical subperiosteal ossification that is separated from the underlying compact bone. Also, ossified soft tissue hemorrhages may occur. In the growing skeleton, scurvy may interfere with the epiphyseal growing zone where irregular micro-fractures produce the radiological Looser zone (19). The long bones remain straight. The skull is usually not affected. A frequent sign is fracture from increased bone fragility.

Since the overall appearance of the infant clearly rules out malnutrition by lack of food, the bone lesions in rickets must have come from another disturbance of vitamin D metabolism. It is interesting that in previous times socially highly ranked people avoided sunlight exposure, and particularly darkening of the skin. Aristocrats were expected to have white, pale skin, whilst laborers were expected to have suntans. This also applied to small infants, who, like the Starhemberg infant, were at risk of developing rickets due to the lack of ultraviolet rays on their skin.

The literature provides only a few cases of infant mummies with the diagnosis of rickets. Panzer et al. (33) described an infant mummy from a Lithuanian crypt with typical radiological evidence. Beyond this, skeletal remains have been investigated for rickets much more intensively (43, 50, 51). These provide extensive data. For example, Mays et al. (50) described the analysis of 15 cemeteries from Roman-period Mediterranean burial sites with 1,143 subadults out of which 5.2% showed skeletal changes consistent with a diagnosis of rickets (*n* = 60). Others described rickets prevalence rates between 13.7 and 48.1% (51). These studies, however, did not relate to social status or any individual identification, except for the study of children from the Medici family discussed above (43).

Finally, the CT scans show residues of the infant's lung with adhesions of the right lung to the chest wall. This observation is highly suggestive of post- or peripleuritic inflammation which is frequently the effect of pneumonia. This may also indicate the cause of death of the individual. Similar features have recently been described in other isolated case reports on infant mummies (20, 24).

Ultimately, there is an aetiological link between rickets and pneumonia that may even exceed the random coincidence of both in historical populations. Recent clinical studies provide clear evidence that children with rickets are much more susceptible to pneumonia (52, 53) where either the reduction in the mechanical properties of the chest wall movement or metabolic features may be the reason. The concurrence of pneumonia with the metabolic bone disorder, which we presume is supportive for both these diagnoses.

### The skull deformation

The skull bones, including the upper cervical spine, showed significant disruption. A meticulous reconstruction of the pieces of the skull bones was undertaken in order to find out the mechanisms of the disruption. The frontal bones were translated under the parietal bone squamae, whilst the skull base was completely disordered. These features might have been the consequence of severe trauma but this typically results in fractures and bleeding. Since we have no evidence of any bleeding such as haematoma formation or skin bruising, direct trauma is unlikely. The bone dislocations were restricted to the skull (and upper cervical spine) but did not affect the rest of the body. It has previously been observed that dehiscence of the coronal suture may occur as ossification variants without any trauma or other manipulations [(54)] occasionally, in mummy collections, such as case no. 75 of the famous Vác mummy collection [Szikossy et al., personal communication], similar dislocations confirm diagenetic pseudopathology and not palaeopathology. It seems most likely that the disruption occurred post-mortem when the child was placed in a flat coffin, too small for the skull.

### Scenario for the reconstruction of the life of the little count of Starhemberg

The likely conclusion is that the mummy is Reichard Wilhelm, 1625–1626 CE, the first son of Erasmus der Jüngere (1595–1664 CE). He had a number of pathological findings where the tentative conclusions are that he was overweight in keeping with being very well fed, had vitamin D deficiency from lack of sunlight resulting in rickets, and that the disruption of his skull bones and upper cervical spine was post-mortem changes from being place in a too flat coffin for the skull. He died aged 10–18 months from pneumonia. His body was wrapped in an expensive silk coat in keeping with his aristocratic status.

## Data availability statement

The original contributions presented in the study are included in the article/supplementary material, further inquiries can be directed to the corresponding author.

## Author contributions

Concept preparation: AN, SP, JW, and OP. Analysis performation: AN, SP, CH, and OP. Preparation of first text draft and preparation of final draft: AN, SP, JW, CH, and OP. All authors contributed to the article and approved the submitted version.

## Conflict of interest

The authors declare that the research was conducted in the absence of any commercial or financial relationships that could be construed as a potential conflict of interest.

## Publisher's note

All claims expressed in this article are solely those of the authors and do not necessarily represent those of their affiliated organizations, or those of the publisher, the editors and the reviewers. Any product that may be evaluated in this article, or claim that may be made by its manufacturer, is not guaranteed or endorsed by the publisher.
